# Electrochemical Oxidation Property of Antioxidative Substances in the Oil-Based Solution

**DOI:** 10.3390/foods15111865

**Published:** 2026-05-25

**Authors:** Guowei Ling, Yu Wang, Mingshuang Xia, Yuhan Yi, Wenlin Li, Shilin Liu, Chengming Wang

**Affiliations:** 1 College of Food Science and Technology, Huazhong Agricultural University, Wuhan 430070, China; lingguowei@webmail.hzau.edu.cn (G.L.); wy0912@webmail.hzau.edu.cn (Y.W.); xms@webmail.hzau.edu.cn (M.X.); yiyuhan@webmail.hzau.edu.cn (Y.Y.); slliu2013@mail.hzau.edu.cn (S.L.); 2 Key Laboratory of Environment Correlative Dietology, Huazhong Agricultural University, Ministry of Education, Wuhan 430070, China; 3 Oil Crops and Lipids Process Technology National & Local Joint Engineering Laboratory, Oil Crops Research Institute of the Chinese Academy of Agricultural Sciences, Wuhan 430062, China; wenlinli2005@163.com

**Keywords:** electrochemical oxidation property, electrochemical characteristic method, radical scavenging capacity, antioxidative substances, oil-based solution, linear sweep voltammetry (LSV)

## Abstract

Antioxidative substances constitute the important barrier maintaining the oxidative stability of edible oils against lipid degradation and the formation of harmful aldehydes and ketones. In this study, an oil-compatible non-aqueous electrochemical method was developed to characterize the oxidation behaviour of antioxidative substances in an oil-based solution, in which linear sweep voltammetry (LSV) was employed to investigate the effects of oxidation potential and current on antioxidative capabilities of eleven antioxidative substances, including both natural and synthetic compounds, in a mixed anhydrous model oil system. Among them, eight antioxidative substances exhibited characteristic oxidation peaks in the mixed solution containing 0.1 mol/L Lithium perchlorate, 40% (*v*/*v*) C8 medium-chain triglyceride, 35% anhydrous ethanol and 25% 1,2-dichloroethane, with the first oxidation peak potentials (vs. Ag/AgCl) increasing in the order: TBHQ (100–800 mg/kg, (−247)–(−119) mV), PG (100–800 mg/kg, 74–248 mV), α-tocopherol (100–800 mg/kg, 95–142 mV), δ-tocopherol (100–850 mg/kg, 190–241 mV), BHT (100–800 mg/kg, 238–256 mV), β-carotene (100–870 mg/kg, 562–624 mV), lutein (100–850 mg/kg, 631–680 mV) and ergosterol (100–850 mg/kg, 1240–1300 mV), while their peak potentials were negatively correlated with the DPPH and Galvinoxyl radical-scavenging capacity, suggesting that, under the present oil-based conditions, lower oxidation peak potentials tended to be associated with stronger radical-scavenging capacity. The concentration–current relationships were compound-dependent and followed linear, cubic, or logarithmic patterns. And the oxidation of phenolic antioxidative substances shifted from a low-potential to a higher-potential process under acidic conditions. Overall, this study reveals the electrochemical oxidation properties of antioxidative substances in the oil-based solution, and provides the electrochemical characteristic method of the antioxidant capacities of antioxidative substances and application guidance for antioxidative substances screening in oil.

## 1. Introduction

In recent years, the addition of antioxidative substances to edible oils has been widely investigated to enhance their stability and extend shelf life [1,2]. Previous studies have demonstrated that appropriate antioxidative substances can markedly improve oxidative stability under thermal processing and storage stress [3]. For example, gallic acid added to sunflower, corn, peanut and hazelnut oils was more effective than butylated hydroxyanisole (BHA) and butylated hydroxytoluene (BHT) in improving oxidative stability [4], and tocopherols and carotenoids can also retard lipid oxidation [5,6]. The oxidative stability of oils is usually characterized using Rancimat and Oxitest methods under accelerated oxidation conditions. However, these conventional tests mainly provide endpoint stability indices and cannot directly characterize the redox behaviour of individual antioxidants in oil. Therefore, an oil-relevant method for evaluating intrinsic antioxidant oxidation properties is still needed.

Electrochemical methods, including cyclic voltammetry, differential pulse voltammetry, square-wave voltammetry, and chronoamperometry, owing to their high sensitivity, rapid response and suitability for complex solution, have been applied to evaluate antioxidant capacity of food such as coffee, tea, rosemary extracts and beverage extracts in aqueous buffer solution by recording oxidation potentials, peak currents, or charge-transfer responses of electroactive antioxidants [7,8]. In organic solution, electrochemical methods can be used to compare antioxidant strength of antioxidative substance. For instance, α-tocopherol with more methyl substituents on the chromanol ring exhibits a lower oxidation potential than δ-tocopherol in acetonitrile solution, indicating that α-tocopherol is more readily oxidized [9]. The voltammetric studies have differentiated the redox behaviour of tocopherol isomers and mapped how solvent, electrode material and medium acidity reshape peak shape and mechanism of oxidation; square-wave voltammetry after the optimization of medium and waveform can simultaneously detect α-, γ- and δ-tocopherol in 0.1 mol/L LiClO_4_ acetonitrile solution [10]. In the oil samples, electrochemical determination of total antioxidant capacity has been achieved in homogeneous ethanol solution through catalytic voltammetric currents generated in an ABTS-based redox probe system [11]. And the synthetic phenolics such as propyl gallate (PG), BHA and BHT have also been quantified well by a voltammetric electronic tongue in edible olive oil sample [12]. Most existing electrochemical approaches rely on aqueous, organic-solvent, or highly diluted alcohol systems, which cannot fully reproduce the low-polarity, viscous, and interfacial environment of oils. Therefore, their electrochemical responses may not accurately represent antioxidant behaviour in oil.

Vegetable oils contain antioxidative substances such as tocopherols, phytosterols and carotenoids. Presently, the representative electrochemical property studies of these antioxidative substances are usually conducted in a 0.1 M LiClO_4_ ethanol/acetonitrile solution [13]. Here, the use of the organic media is essential, because the organic phase improves the solubility of lipophilic antioxidative substances and enables a clearer characterization of the electrochemical electron-transfer and deprotonation processes of antioxidative substances [14,15]. However, vegetable oils are characterized by low polarity, relatively high viscosity, and distinct interfacial properties, affecting electrochemical oxidative intermediate stability, the extent of proton coupling and mass-transfer rate of antioxidative substance. As demonstrated by the experiments conducted, antioxidative substances in pure vegetable oil such as soybean oil, peanut oil, flaxseed oil, camellia oil, and C8 medium-chain triglyceride oil containing 0.1 mol/L lithium perchlorate electrolyte failed to produce clear electrochemical oxidation signals, owing to the inherently low conductivity of the medium and the slow mass transfer resulting from the high viscosity of vegetable oil. To better mimic the electrochemical oxidation property of antioxidative substances in vegetable oils, a mixed solution consisting of vegetable oil and organic solvent was employed as the electrochemical oxidation environment for antioxidative substance.

In this work, a clean oil without antioxidative substance was first screened, and an anhydrous, blended non-aqueous model oil medium was employed to obtain electrochemical oxidation parameters, such as peak potential and peak current, of eleven antioxidative substances (α/δ-tocopherol, lutein, β-carotene, ergosterol, stigmasterol, β-sitosterol, squalene, PG, BHT and TBHQ), where peak potential was used as an empirical indicator of oxidation tendency under the present oil-based conditions, while peak current was used to describe the oxidation response intensity and its quantitative potential. Furthermore, the peak current–concentration relationships of the antioxidative substances and the difference in their electrochemical oxidation were studied in the oil-based solution, and the acidic concentration dependence of the electrochemical oxidation of the antioxidative substances was elucidated. In addition, the electrochemical parameters of the antioxidative substances were linked to their free-radical scavenging performance. These studies will provide applied guidance for the antioxidative substances to improve the oxidative stability of vegetable oils.

## 2. Materials and Methods

### 2.1. Materials and Reagents

C8 medium-chain triacylglycerol (C8 MCT oil) was supplied by Sports Research Corporation (San Pedro, CA, USA). Glyceryl trioleate was purchased from Sinopharm Chemical Reagent Co., Ltd. (Shanghai, China). α-Tocopherol standard (>96%), BHT (>99%), and PG (98%) were purchased from Shanghai Macklin Biochemical Co., Ltd. (Shanghai, China). δ-Tocopherol standard (>90%), stigmasterol (>95%), β-sitosterol (>95%), ergosterol (>95%), lutein (>80% HPLC), and β-carotene (>97%) were purchased from Shanghai Yuanye Bio-Technology Co., Ltd. (Shanghai, China). TBHQ (>98%) was purchased from Shanghai Aladdin Bio-Chem Technology Co., Ltd. (Shanghai, China). Methanol, n-hexane, and the derivatization reagent (99% BSTFA + 1% TMCS) were of chromatographic grade and were obtained from Shanghai Macklin Biochemical Co., Ltd. (Shanghai, China). 1,2-Dichloroethane was obtained from Shandong Keyuan Technology Co., Ltd. (Laizhou, China). Other reagents, including anhydrous ethanol (purity ≥ 99%), concentrated sulfuric acid (purity ≥ 98%), and potassium hydroxide, were of analytical grade and were purchased from Shanghai Chemical Reagent Co., Ltd. (Shanghai, China). Deuterated chloroform was purchased from Shanghai Macklin Biochemical Co., Ltd. (Shanghai, China), and dimethyl terephthalate was obtained from Shanghai Aladdin Bio-Chem Technology Co., Ltd. (Shanghai, China). Soybean oil, peanut oil, and flaxseed oil were purchased from Yihai Kerry Arawana Holdings Co., Ltd. (Shanghai, China). Camellia oil was purchased from Zou Youcai Food Co., Ltd. (Harbin, China).

### 2.2. Purification of Vegetable Oil

In this study, the commercial vegetable oils (soybean, peanut, flaxseed, and camellia) were purified using a 30 × 500 mm chromatographic column packed from the bottom to top with silica gel (300–400 mesh size), activated carbon, and alumina (200–300 mesh size) at mass ratios of 1:0.5:1, 1:0.5:1.5, and 1:0.5:2, respectively, referring to the previous method [16].

### 2.3. Determination of Tocopherols by Ultra Performance Liquid Chromatography (UPLC)

The content of tocopherols was determined with reference to the previous studies [17]. Oil samples (0.2 g) were dissolved in 2 mL n-hexane, and the mixture was withdrawn with a syringe and filtered through a 0.22 μm organic membrane filter prior to analysis. A total of 20 μL of the filtered samples was injected into the UPLC system Ultimate 3000; Thermo Fisher Scientific, Waltham, MA, USA) equipped with an Acclaim™ C30 column (250 mm × 4.6 mm, 3 μm; Thermo Fisher Scientific, Waltham, MA, USA). Methanol was used as the mobile phase at a flow rate of 0.80 mL/min, and detection was performed at a wavelength of 294 nm using an ultraviolet detector (UVD). The various tocopherol content was quantified based on the calibration curves constructed from a series of methanol solutions containing α-, γ-, and δ-tocopherols with concentrations of 1.0, 2.5, 5.0, 10.0, 25.0, 50.0, and 100.0 μg/mL, with results expressed as mg/kg of oil, and the standard curves of α-, γ-, and δ-tocopherols are as follows: α-tocopherol: y = 0.0992x + 0.0159, R^2^ = 0.9997; β-tocopherol:y = 0.1414x − 0.0559, R^2^ = 0.9997; γ-tocopherol: y = 0.2006x + 0.0414, R^2^ = 0.9997; δ-tocopherol: y = 0.1412x − 0.0195, R^2^ = 0.9999.

### 2.4. Determination of Phytosterols by Gas Chromatography

The content of phytosterols was determined according to the methods of previous studies [18,19]. An accurately weighed sample (250 mg, to the nearest 1 mg) was placed in a 10 mL centrifuge tube. Then, 200 μL of a 0.1% cholesterol internal standard solution was added, followed by 2.0 mL of 0.5 mol/L KOH in ethanol. The tube was vigorously shaken for a few seconds and incubated in an 80 °C water bath for 30 min, with vortexing every 10 min. After cooling to room temperature, 1.0 mL n-hexane was added for organic extraction of the unsaponifiable fraction, and 1.5 mL water was then added to achieve clear phase separation. The tube was shaken vigorously and centrifuged at 4000 rpm for 10 min. The upper hexane layer (containing free phytosterols) was transferred to a new plastic tube. This extraction was repeated three times, and the collected extracts were combined and dried under a gentle nitrogen stream. After drying, 150 μL of the derivatisation reagent BSTFA:TMCS (99:1, *v*/*v*) was added. The mixture was vortexed for 15 s and reacted in a 70 °C water bath for 30 min. After filtration through a 0.22 μm organic membrane filter, 1 μL of the filtrate was injected using a microsyringe into a gas chromatograph (6890N; Agilent Technologies, Santa Clara, CA, USA) equipped with an HP-5MS column (30 m × 0.25 mm × 0.25 μm; Agilent Technologies, Santa Clara, CA, USA) for analysis. The initial column temperature was set at 200 °C and held for 3 min, then increased to 220 °C at a rate of 2 °C/min, followed by a further increase to 300 °C at a rate of 20 °C/min and held for 15 min. The injector and detector temperatures were maintained at 300 °C. Nitrogen was used as the carrier gas at a flow rate of 1.0 mL/min, and the sample split ratio was 15:1. Based on the following equation, phytosterols and squalene were quantified using cholesterol as an internal standard substance, with the results being expressed as milligrams of phytosterols or squalene per 100 g of oil.

$$S=\frac{A_{0}\times m_{i}}{A_{i}\times m_{T}}\times 100$$

where S is the content of phytosterol or squalene in the sample, the unit is mg/100 g; A_0_ is the peak area of phytosterol or squalene in the sample; m_i_ is the mass of internal standard cholesterol in the sample, m_i_ = 0.2 mg; A_i_ is the peak area of the internal standard cholesterol added to the sample; and m_T_ is the mass of the sample, the unit is g.

### 2.5. Determination of Carotenoids and Chlorophyll by Spectrophotometer

According to the method of Pohndorf (2020) [20], 1.000 g of oil sample (accurate to 0.001 g) was accurately weighed, dissolved in n-hexane, and diluted to 10.0 mL. The absorbance was measured at 446, 630, 670, and 710 nm using the UV-1800 UV–Vis spectrophotometer (Shimadzu Corporation, Kyoto, Japan). Carotenoid and chlorophyll contents were calculated using the following equations:

$$\mathrm{Carotenoid}\;\mathrm{content}\;(\mathrm{mg}/\mathrm{kg}):=\frac{383A_{446}}{\mathrm{mc}}$$

$$\mathrm{chlorophyll}\;\mathrm{content}\;(\mathrm{mg}/\mathrm{kg})=\frac{\left(A_{670}-\frac{A_{630}+A_{710}}{2}\right)V}{0.0964m}$$

where m is the sample mass (g), c is the concentration of the diluted sample (g/100 mL), 383 is the extinction coefficient of carotenoids, and V is the final volume after dilution.

### 2.6. Pre-Test Preparation

Electrochemical measurements were performed in a three-electrode configuration using a glassy carbon electrode as the working electrode, an Ag/AgCl electrode as the reference electrode, and a platinum wire electrode as the counter electrode. The electrode pretreatment followed the procedure reported previously [21]. The working electrode was successively ground, polished and activated in 0.5 M H_2_SO_4_ for 30 min, and then stored for use. After pretreatment, the electrode performance should satisfy that the peak potential interval (ΔE_p_) between the oxidation and reduction peaks is less than 100 mV, and the oxidation and reduction peak current ratio is 1 by cyclic voltammetry in 1 mM potassium ferricyanide solution [22].

The electrolyte concentration and organic solvent type were preliminarily screened based on the electrochemical peak profiles and safety considerations. Exactly 0.106 g of lithium perchlorate was weighed and dissolved in 3.5 mL ethanol. Then, 2.5 mL 1,2-dichloroethane and 4.0 mL C8 MCT oil were added to the cell. The mixture was transferred into a 50 mL electrochemical cell. The base solution was deoxygenated by purging with nitrogen for 2 min and kept under a continuous nitrogen flow from a gas bag during measurements to maintain an oxygen-free environment. Linear sweep voltammograms were recorded at a scan rate of 100 mV s^−1^, and all experiments were conducted under dim-light conditions to avoid photo-induced effects.

### 2.7. Electrochemical Characterization of Antioxidative Substances in the Oil-Based Solution

The base solution was spiked with the given amounts of antioxidative substances (such as α/δ-tocopherols and carotenoids) to prepare standard solutions at the desired concentrations. The three electrodes were inserted into the electrochemical cell, and nitrogen was gently purged. CV was first used to observe the reversibility of the redox reactions, followed by LSV to measure the peak potentials and peak currents of oxidation. The potential scan ranges in CV and LSV were adjusted according to the peak positions of the antioxidative substances. Mass concentration was retained because mg/kg is commonly used for antioxidant addition in edible oils. To support mechanistic interpretation, the corresponding molar-equivalent concentrations were calculated from the molecular weight of each compound and added to Table 1 and Table 2.

### 2.8. Antioxidant Testing

#### 2.8.1. DPPH Radical-Scavenging Assay

DPPH radical scavenging activity was determined according to the method of previous studies [23]. Accurately weigh 1 mg of DPPH standard, dissolve in anhydrous ethanol, and make up to 25 mL to prepare a 0.1 mmol/L DPPH solution. In a 96-well plate, add 100 μL of antioxidative substance sample and 100 μL of the DPPH solution, react for 30 min at room temperature in the dark, and measure the absorbance A_1_ at 517 nm to calculate the DPPH free-radical scavenging rate.

DPPH free-radical scavenging rate (%) = [1 − (A_1_ − A_2_)/A_0_] × 100%

where A_0_ is the absorbance of the control without sample; A_1_ is the absorbance of the mixture of sample solution and DPPH solution; and A_2_ is the absorbance of the mixture of anhydrous ethanol and the sample solution.

#### 2.8.2. Galvinoxyl Radical-Scavenging Assay

Galvinoxyl radical scavenging activity was determined according to the method of previous studies [24]. Accurately weigh 1 mg of galvinoxyl standard, dissolve in n-hexane, and make up to 25 mL to obtain a 0.1 mmol/L stock solution. In a 96-well plate, add 100 μL of sample and 100 μL of the galvinoxyl solution to each well. Incubate in the dark at room temperature for 30 min, then measure the absorbance at 428 nm (A_1_). Calculate the galvinoxyl radical-scavenging rate as:

Galvinoxyl free-radical scavenging rate (%) = [1 − (A_1_ – A_2_)/A_0_] × 100%

where A_1_ is the absorbance of the mixture of sample solution and galvinoxyl solution; A_0_ is the absorbance of anhydrous ethanol mixed with galvinoxyl solution (blank control); and A_2_ is the absorbance of the mixture of sample solution and anhydrous ethanol.

### 2.9. Quantitative ^1^H NMR Determination

^1^H NMR spectra were recorded on a fully digital superconducting NMR spectrometer (Bruker AVANCE III 600 MHz NMR spectrometer (Bruker BioSpin GmbH, Rheinstetten, Germany)). For sample preparation, 500 μL of the sample solution was mixed with 100 μL of an internal standard solution containing 2 mg/mL dimethyl terephthalate (DMT). The mixture was evaporated to dryness under a gentle stream of nitrogen to remove the organic solvents, and the residue was then redissolved in 300 μL of deuterated chloroform (CDCl_3_). The resulting solution was finally transferred into a 5 mm NMR tube for analysis. The ^1^H NMR measurements were performed using a 5 mm BBO broadband observe probe (resolution, R ≤ 0.60 Hz; sensitivity, S/N ≥ 900:1). The acquisition parameters were set as follows: 30° pulse angle, 298 K, 16 scans (NS), and a relaxation delay (D1) of 5 s. Before data acquisition, all samples were pre-cooled in an ice pack containing saturated NaCl solution and protected from light. Quantitative analysis was carried out by comparing the integral area of the target resonance with that of the internal standard, because the signal integral is proportional to the molar amount of the corresponding protons [25].

### 2.10. Fourier Transform Infrared (FTIR) Spectroscopy Determination

Fourier transform infrared (FTIR) spectra were recorded on a Nicolet iS50 FTIR spectrometer (Thermo Fisher Scientific, Waltham, MA, USA) to characterize the functional groups and structural features of antioxidative substances in liquid samples. An oil-based solution (without the target antioxidative substances) was collected as the background spectrum, and sample spectra were background-corrected accordingly. Spectra were acquired over 4000–400 cm^−1^ with a resolution of 4 cm^−1^ and 64 co-added scans to improve the signal-to-noise ratio. The spectra were reported in transmittance mode (%T) with a data spacing of 0.482 cm^−1^.

### 2.11. Statistical Analysis

All analyses were performed in triplicate, and the results are expressed as mean ± standard deviation (SD). Data processing and regression analysis were conducted using SPSS software (version 25.0, IBM Corp., Armonk, NY, USA). The relationships between concentration and peak current were fitted using least-squares regression, and the goodness of fit was evaluated using the coefficient of determination (R^2^). The 95% confidence and prediction bands were calculated for the corresponding calibration curves where applicable.

## 3. Results and Discussion

### 3.1. Selection of the Model Oil

Through gas chromatography and high-performance liquid chromatography analyses, the results showed that the vegetable oils after column purification and the glyceryl trioleate still contained tocopherols and sterols. Their contents were 110 mg/kg and 52 mg/100 g in soybean oil, respectively; 6 mg/kg and 100 mg/100 g in peanut oil, respectively; 44 mg/kg and 180 mg/100 g in flaxseed oil, respectively; not detected and 160 mg/100 g in camellia oil, respectively; 320 mg/kg and 240 mg/100 g in glyceryl trioleate, respectively, whereas the endogenous antioxidative substances such as tocopherols, sterols, carotenoids and squalene were not detected in C8 MCT oil, maybe because the endogenous antioxidative substances are easily isolated from C8 MCT oil. Moreover, C8 MCT oil is a fully saturated triacylglycerol with low susceptibility to autoxidation and oxidation by-product formation [26]. Therefore, C8 MCT oil was subsequently selected as the model oil.

### 3.2. Effects of Concentration on the Electrochemical Oxidation of Antioxidative Substances in the Oil-Based Solution

**Figure 1:**
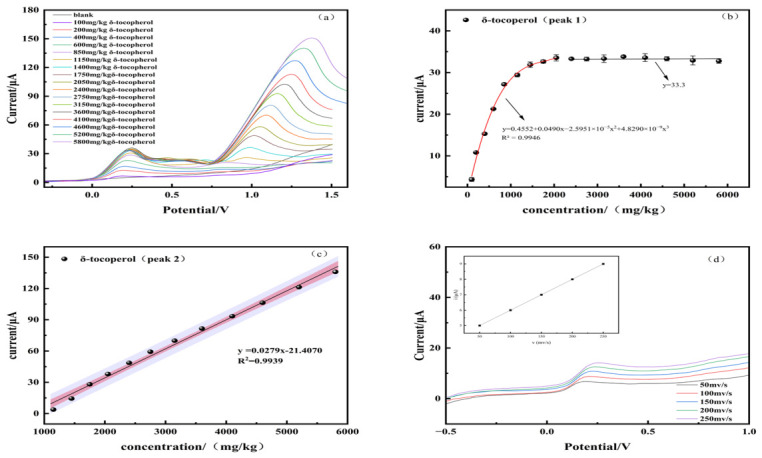
Linear sweep voltammograms (LSVs) of δ-tocopherol at different concentrations in the oil-based solution. (**a**) LSVs at different δ-tocopherol concentrations. (**b**) Fitting curve between concentration and the first oxidation peak current. (**c**) Fitting curve between concentration and the second oxidation peak current, with the red and blue shaded regions representing the 95% confidence and prediction bands, respectively. (**d**) LSVs of 200 mg/kg δ-tocopherol at different scan rates.

**Figure 2:**
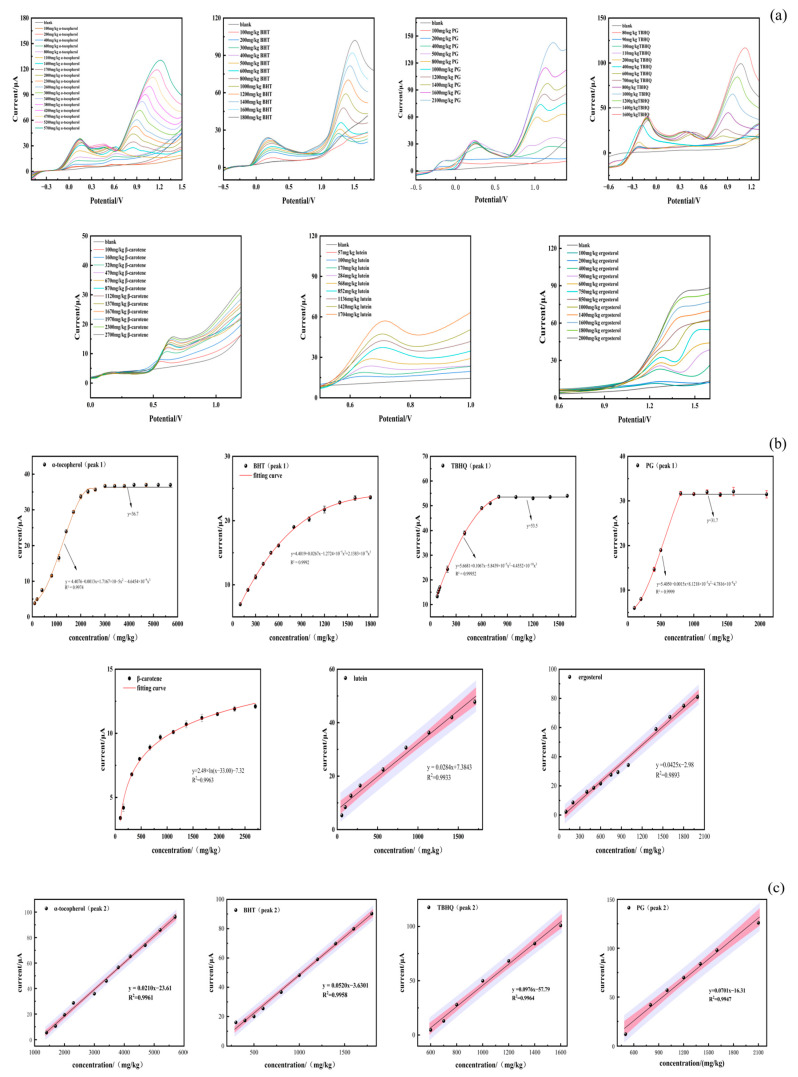
Linear sweep voltammograms (LSVs) of α-tocopherol, BHT, TBHQ, PG, β-carotene, lutein, and ergosterol at different concentrations in the oil-based solution (**a**). The corresponding calibration plots are shown in (**b**,**c**) for the first and second oxidation peaks, respectively; β-carotene, lutein, and ergosterol exhibited only one oxidation peak. The red and blue shaded regions represent the 95% confidence and prediction bands, respectively.

The electrochemical oxidation of an antioxidative substance in the organic solvents such as benzene and ethanol aqueous solutions is strongly dependent on its bulk concentration, which determines its coverage and diffusion layer thickness on the electrode surface, as well as the appearance or disappearance of secondary oxidation pathways, e.g., dimerization or quinone formation of phenolic compounds as antioxidative substances [27]. To characterize the electrochemical oxidation behaviour of antioxidative substances in the oil-based solution, δ-tocopherol was first selected as a representative compound, and its LSV response is shown in Figure 1. The results showed that the detection limit of δ-tocopherol in a 0.1 mol/L lithium perchlorate oil-based solution was around 40 mg/kg, whereas the detection limit of δ-tocopherol in 0.1 mol/L lithium perchlorate acetonitrile solution was 0.49 mg/kg [10], indicating that the detection limit of antioxidative substances in the oil-based solution is much higher than that in the organic solution. Moreover, as shown in Figure 1a, the electrochemical oxidation potential and peak current of δ-tocopherol in the oil-based solution are closely related to its concentration. At a concentration as low as 100 mg/kg, a single oxidation peak of δ-tocopherol appears at around 190 mV, corresponding to the initial one-electron oxidation of the phenolic OH group, maybe forming a phenoxyl radical [28]. As the concentration of δ-tocopherol increases from 100 to 2050 mg/kg, the initial oxidation peak potential increased from 190 to 248 mV (Figure 1a), and the peak current increased from 4.3 to 33 μA. As the concentration further increased to 5800 mg/kg, the initial oxidation peak potential instead decreased to 231 mV, and the peak current maintained 33 μA. The concentration–peak current curve relationship ranging from 100 to 2050 mg/kg fitted with a cubic polynomial function, y = 0.4552 + 0.0490x − 2.5951 × 10^−5^x^2^ + 4.8290 × 10^−9^x^3^, R^2^ = 0.9946, where y is the peak current, x is the concentration of δ-tocopherol (Figure 1b), because the adsorption of δ-tocopherol on the electrode surface approaches saturation at the high concentrations from 2050 to 5800 mg/kg so that the mass transfer of δ-tocopherol becomes diffusion-limited (Nernst layer), resulting in a plateau of the peak current [29,30]. Similarly, the low-potential oxidation peak currents of phenolic antioxidative substances such as α-tocopherol, PG and TBHQ also reached stable currents at appropriate concentrations after their oxidation peak current increased with an increase in the concentration (α-tocopherol, y = 4.4076 − 0.0013x + 1.7167 × 10^−5^x^2^ − 4.6454 × 10^−9^x^3^, R^2^ = 0.9974, 100–2600 mg/kg; PG, y = 5.40501 − 0.0015x + 8.1218 × 10^−5^x^2^ − 4.7816 × 10^−8^x^3^, R^2^ = 0.9999, 100–800 mg/kg; TBHQ, y = 5.6681 + 0.10671x − 5.8439 × 10^−5^x^2^ − 4.4552 × 10^−10^x^3^, R^2^ = 0.9995, 80–800 mg/kg) (Figure 2). For BHT, the peak current increased gradually with concentration from 100 to 1800 mg/kg and was fitted by y = 4.4019 + 0.0267x − 1.2724 × 10^−5^x^2^ + 2.1383 × 10^−9^x^3^ (R^2^ = 0.9992). At 1600–1800 mg/kg, the peak current gradually approached a plateau.

**Figure 3:**
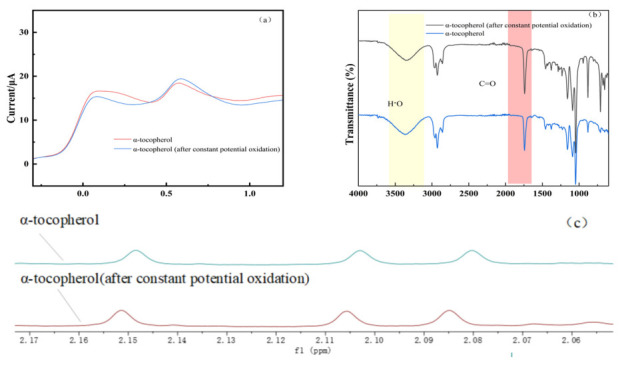
LSV voltammogram (**a**), IR spectrum (**b**), and ^1^H NMR spectrum (**c**) of 800 mg/kg α-tocopherol with or without 12 h of constant-potential electrolysis.

Simultaneously, a second distinct oxidation peak of the five phenolic antioxidative substances emerges. For δ-tocopherol, the second distinct oxidation peak emerges at around 971 mV when the concentration is increased to 1150 mg/kg, and its potential also shows a progressively positive shift with increasing concentration (Figure 1a); the peak current of the second peak exhibits a linear relationship with concentration (Figure 1c). For the other four phenolic compounds such as α-tocopherol, PG, TBHQ, BHT, their potential of the second oxidation peak also shows a progressively positive shift with increasing concentration (Figure 2), and their peak current of the second peak exhibits a linear relationship with their concentrations (Figure 1c), whereas no second oxidation peak was observed for the carotenoids or ergosterol (Figure 2). It is reported that α-tocopherol as a phenolic compound in aqueous solution is oxidized using cyclic voltammetry to form a quinone/quinone–hydroquinone reversible couple [31], and the electro-oxidation of BHT in acetonitrile–aqueous solution using cyclic voltammetry can yield a dimer [32]. In this study, α-tocopherol in the oil-based solution after continuous potentiostatic electrochemical oxidation at 107 mV and 565 mV corresponding to its potentials of two oxidation peaks for 12 h, was measured using LSV, FTIR and ^1^H NMR spectra, in a comparison with the responses before and after electrochemical treatment. The results showed that, after the electrochemical treatment, the initial oxidation peak current of α-tocopherol decreased while the second oxidation peak current of α-tocopherol increased (Figure 3a), indicating that the controlled-potential oxidation preconverts a portion of α-tocopherol (TOH), making α-tocopherol decrease, into its products for further oxidation, making the initial oxidation production α-tocopherol increase. Simultaneously, the absorption peak of O–H band of α-tocopherol at 3200–3600 cm^−1^ was weakened and the absorption peak of C=O band at 1650–1750 cm^−1^ was enhanced in the FTIR spectra (Figure 3b), whereas the integral of the aromatic methyl protons at 2.06–2.16 ppm remained unchanged and no new signals appeared in the ^1^H NMR spectra (Figure 3c). These results indicate that α-tocopherol was mainly oxidized to quinone/hydroquinone-type products, as supported by the FTIR spectra, while the likelihood of extensive polymer formation was low based on the ^1^H NMR spectra. Similarly, these findings provide a basis for interpreting the electrochemical oxidation characteristics of other phenolic antioxidants, such as PG, BHT, and TBHQ.

For β-carotene, lutein and ergosterol, only one oxidation peak was observed over their tested concentration ranges. Among them, the peak currents of lutein and ergosterol increased linearly with an increase in concentration; the corresponding equations were fitted as follows: for lutein, y = 0.0284x + 7.3843, (R^2^ = 0.9933, 57–1704 mg/kg), and for ergosterol, y = 0.0425x − 2.98, (R^2^ = 0.9893, 100–2000 mg/kg), whereas the peak currents of β-carotene increased logarithmically with an increase in concentration (y = 2.49 × ln(x−33.006) − 7.32, (R^2^ = 0.9963, 100–2700 mg/kg)), indicating that lutein and ergosterol exhibit mainly diffusion-controlled oxidation in the test concentration ranges, because they are present as well-solubilized substances in the oil-based solution, whereas β-carotene is more prone to aggregation and interfacial adsorption in the oil-based solution, causing progressive surface saturation and a decrease in the effective concentration at the electrode surface, thereby resulting in a logarithmic response [33]. In contrast, stigmasterol, β-sitosterol, and squalene showed no observable oxidation peak, indicating that β-Sitosterol and stigmasterol exhibit weak direct electroactivity, whereas squalene lacks strongly electroactive functional groups [34,35]. Moreover, under the low-conductivity and slow mass-transfer conditions of the oil-based solution, their oxidation responses were markedly suppressed, and thus no obvious oxidation peaks were observed.

### 3.3. Comparation of the Electrochemical Properties of Antioxidative Substances in the Oil-Based Solution

The electrochemical parameters of different antioxidative substances in the oil-based solution from their linear scanning voltammogram were summarized in Table 1 and Table 2. At the same practical mass concentration of 100 mg/kg, the first oxidation peak potentials followed the order TBHQ (−247 mV) < PG (74 mV) < α-tocopherol (95 mV) < δ-tocopherol (191 mV) < BHT (256 mV) < β-carotene (562 mV) < lutein (631 mV) < ergosterol (1240 mV). Because the corresponding molar concentrations differ among compounds, this comparison was used mainly to evaluate structure-related oxidation tendency rather than identical molar electrochemical response. In the present oil-based system, a lower peak potential generally indicates easier oxidation and tends to be associated with stronger radical-scavenging performance. This sequence is highly consistent with the number of phenolic hydroxyl groups, the electron-donating ability of aromatic substituents, and the resonance stabilization of the corresponding oxidized radicals. TBHQ possesses a p-diphenol skeleton with two tert-butyl groups, and the resulting p-diphenoxyl-type radical upon proton-coupled electron transfer (PCET) oxidation is extensively delocalized over the aromatic ring and further stabilized by the strongly electron-donating tert-butyl groups, making TBHQ the easiest to be oxidized so as to give the lowest potential [36]. Although PG contains three phenolic hydroxyl groups, the aromatic ring is substituted with an electron-withdrawing ester group, which slightly reduces the π-electron density compared with TBHQ, leading to a somewhat more positive potential. α- and δ-tocopherol share the same chromanol ring, but the α-isomer carries three methyl groups whereas δ-tocopherol has only one, so the electron-donating effect decreases in the order α-tocopherol > δ-tocopherol, and the oxidation potentials follow α-tocopherol < δ-tocopherol. BHT has only one phenolic hydroxyl group, which is sterically shielded by two bulky tert-butyl groups; the O–H acidity and PCET capability are therefore weaker, leading to a more positive peak potential. In contrast, β-carotene and lutein possess long-range π-conjugation that can partially stabilize positive charge [37], but they lack phenolic hydroxyl groups and participate in oxidation mainly via electron transfer along the conjugated polyene chain [38], resulting in markedly more positive peak potentials. Ergosterol contains only a conjugation and no phenolic hydroxyl group, and its oxidized products are difficult to reach resonance stabilization; therefore, it exhibits the most positive oxidation peak potential [39].

Table 2 summarizes the electrochemical parameters of the second oxidation peak for the above phenolic antioxidative substances such as α-tocopherol, δ-tocopherol, BHT, PG and TBHQ. The results showed that the second oxidation peak does not appear at the same concentration for all compounds, but instead shows a system-dependent “threshold”: the second peak becomes observable for α-tocopherol at around 1400 mg/kg, for δ-tocopherol at 1150 mg/kg, and for BHT, PG and TBHQ already at approximately 300, 400 and 600 mg/kg, respectively, indicating that the secondary oxidation of these phenolic compounds requires different concentrations, namely, only when the phenoxyl radicals generated in the first oxidation step reach sufficiently high concentrations at the electrode interface of the solution, and undergo quinone-forming reactions. The phenoxyl radicals are quinonoid products produced in amounts large enough to be re-oxidized at more positive potentials, indicating that α- and δ- tocopherols have the lower effective enrichment at the oil–electrode interface, maybe because they possessing a long hydrophobic side chain diffuse more slowly, similarly to phenolic acids in aqueous solution [40]. In contrast, BHT, PG, and TBHQ with smaller side chains can accumulate sufficient secondary products even at lower concentrations. For squalene, β-sitosterol, and campesterol from 100 to 10,000 mg/kg, respectively, there is no characteristic oxidation peaks in the LSV curves scanning from −5 to 5 V, indicating that these three compounds could not be electrochemically oxided in the oil-based solution, because their C=C bonds in the molecular structures are not conjugated.

At 1400 mg/kg as a comparable concentration in Table 2, PG, TBHQ, and BHT delivered markedly larger peak currents than the tocopherols (83, 84, and 70 μA vs. 5 μA for α-tocopherol and 14 μA for δ-tocopherol), indicating that PG, TBHQ, and BHT retain stronger antioxidative capability than α-tocopherol and δ-tocopherol during the second oxidation step. Because peak current reflects the efficiency of electron transfer in the process of oxidation [32,41], the potential order of the second oxidation peak, following α-tocopherol (841 mV) < δ-tocopherol (990 mV) < TBHQ (1062 mV) < PG (1128 mV) < BHT (1439 mV) at 1400 mg/kg (Table 2), differs from that of the first peak at 100 mg/kg, indicating that the antioxidation capacity of the antioxidative substances is typically influenced by both oxidation peak potential and peak current. And the potential of the second peak of the five phenolic compounds increased with increasing concentration (Figure 2a), consistent with that of δ-tocopherol. When the concentrations of PG, TBHQ, BHT, α-tocopherol and δ-tocopherol were changed (Table 2), the peak current of every antioxidative substance scaled linearly with concentration (R^2^ > 0.99). The slopes of the calibration plots followed TBHQ > PG > BHT > δ-tocopherol > α-tocopherol, indicating that TBHQ and PG remain highly electroactive at the second oxidation step beyond the first oxidation, whereas the tocopherols, especially α-tocopherol, show a markedly attenuated second-stage response, suggesting α-tocopherol single-radical capture propensity.

### 3.4. Effects of Scan Rate on the Electrochemical Oxidation of Antioxidative Substances in the Oil-Based Solution

The effect of scan rate on δ-tocopherol was examined to elucidate the relationships of peak current and peak potential with scan rate, thereby clarifying the rate-controlling step and electron-transfer characteristics of its oxidation process in the oil-based solution. In Figure 1d, the oxidation peak current of δ-tocopherol increases linearly with an increase in scan rate from 50 to 250 mV·s^−1^, while its peak potential shows a slight positive shift. Such behaviour is consistent with a quasi-adsorption-controlled surface process rather than bulk diffusion control, which may be attributed to hydrophobic interactions of δ-tocopherol in the oil-based solution with the surface of the glassy carbon electrode and π–π interactions between δ-tocopherol and the surface of the glassy carbon electrode lead to interfacial enrichment of δ-tocopherol at the electrode, resulting in an approximately linear relationship between the peak current and scan rate over the scan rate range, which is consistent with the phenomenon of diosmin as a phenolic compound in the aqueous solution [42]; meanwhile, the small positive shift in the oxidation potential of δ-tocopherol appeared with an increase in scan rate, maybe because of the quasi-irreversibility of charge transfer, together with ohmic potential drop and kinetic polarization, which is consistent with the phenomenon of a ferrocene derivative as an antioxidative substance in the aqueous solution [43].

### 3.5. Free-Radical Scavenging Rate of Antioxidative Substances in the Oil-Based Solution

In conventional Trolox equivalent antioxidant capacity (TEAC) protocols, ABTS•^+^ is generally prepared and measured in distilled water or aqueous buffer. However, the present oil-based solution is immiscible with water so as to mass-transfer limitations and optical scattering, which may result in artefactual scavenging readouts. Likewise, the assays of superoxide anion scavenging, hydroxyl radical scavenging and oxygen radical absorbance capacity, which also require aqueous media, are not recommended for direct application to this oil-based solution. In the oil-based solution employed herein, assays of DPPH and galvinoxyl are suitable for free-radical scavenging rate.

**Figure 4:**
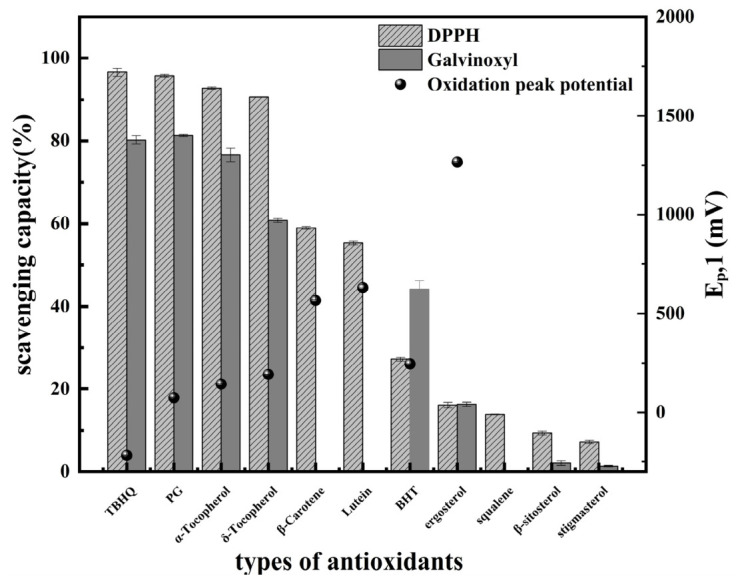
Summary of the radical scavenging rates (DPPH and Galvinoxyl) and initial peak potentials of 11 antioxidative substances (50 μg/mL) dissolved in the oil-based solution. Data are expressed as mean (*n* = 3) ± standard deviation.

In this study, the antioxidant capacities of the antioxidative substances in the oil-based solution were evaluated using DPPH and galvinoxyl radical scavenging assays. As shown in Figure 4, the DPPH radical scavenging rate of a 50 μg/mL solution followed the order: TBHQ (96.63 ± 0.96%) > PG (95.76 ± 0.31%) > α-tocopherol (92.8 ± 0.28%) > δ-tocopherol (90.68 ± 0.02%) > β-carotene (59 ± 0.34%) > lutein (55.4 ± 0.42%) > BHT (27.24 ± 0.41%) > ergosterol (16.1 ± 0.66%) > squalene (13.9 ± 0.10%) > β-sitosterol (9.4 ± 0.42%) > campesterol (7.2 ± 0.42%). Their order was identical to the first oxidation peak potentials in the LSV scans except BHT. This consistency indicates that LSV peak potential and radical-scavenging activity are governed by a common electron-donation mechanism: compounds oxidized at lower potentials usually quench radicals more effectively because their oxidized intermediates are stabilized by phenolic hydroxyl groups, aromatic resonance, or conjugated structures. Although BHT showed a lower oxidation potential than carotenoids in the LSV measurements, its radical scavenging activity was not correspondingly stronger. This can be attributed to the steric hindrance and slower reaction kinetics of BHT with DPPH radical, similarly to that in methanol solution [44], leading to a relatively lower scavenging efficiency. The overall scavenging rates of the galvinoxyl radical for the 50 μg/mL solution were lower than those observed in the DPPH assay except BHT, in which the galvinoxyl radical is not suitable for evaluating the antioxidant activities of β-carotene, lutein and squalene, mainly because β-carotene, lutein, and squalene absorb at 428 nm, the detection wavelength of galvinoxyl radical, which led to abnormally low or even negative scavenging rates. And the ranking of galvinoxyl radical scavenging activity is consistent with that obtained from the DPPH assay, indicating that the resulting order of radical scavenging abilities from the galvinoxyl and DPPH assays in the solution remains comparable although the two radicals follow different reaction pathways [45,46].

For squalene, β-sitosterol, and stigmasterol, the tested concentration range was 100–10,000 mg/kg. The DPPH radical scavenging rates of the three compounds were 13.9%, 9.4%, and 7.2%, respectively, while their corresponding galvinoxyl radical scavenging rates were undetectable, 2.0%, and 1.4%, respectively, indicating that these substances have low radical scavenging capacity, because their C=C bonds in the molecular structures are not conjugated.

### 3.6. Effects of Acid and Base Levels on the Electrochemical Oxidation of Antioxidative Substances in the Oil-Based Solution

**Figure 5:**
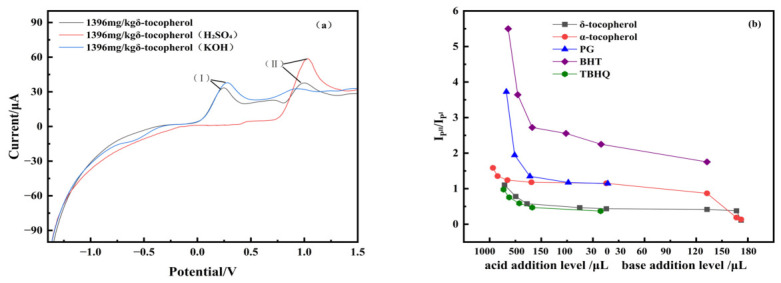
Effect of acid/base addition on the electrochemical oxidation behavior of phenolic antioxidants in the oil-based solution. (**a**) Voltammograms of 1396 mg/kg δ-tocopherol under neutral, acidic, and alkaline conditions; peak I and peak II denote the first low-potential oxidation peak and the second high-potential oxidation peak, respectively. (**b**) Ratio of the peak current of peak II to that of peak I under different acid/base addition levels for 1600 mg/kg δ-tocopherol, 3000 mg/kg α-tocopherol, 800 mg/kg PG, 800 mg/kg BHT, and 800 mg/kg TBHQ. The acidic and basic solutions were 10 mmol/L H_2_SO_4_ in ethanol and 10 mmol/L KOH in ethanol, respectively.

In vegetable oils, free fatty acids, refining additive acid/base, and small amounts of water can change the effective acidity/proton-donor availability of the oil phase. Acid/base conditions regulate proton availability and deprotonation of antioxidative substances, thereby affecting the voltammetric parameters (peak potentials, peak shapes, and peak currents of phenolic antioxidants [28]. Therefore, it is necessary to evaluate how acid/base dosage modulates the electrochemical responses and mechanisms of antioxidants in the oil-based solution. Figure 5 clearly demonstrates the influence of acid/base addition levels on the electrochemical oxidation of phenolic antioxidative substances in the oil-based solution. In Figure 5a, using δ-tocopherol in the oil-based solution as a representative phenol example, oxidation peak I without any added acid or base appeared at ~243 mV with a peak current of 31 μA, while its oxidation peak II was observed at ~1000 mV with a peak current of 17 μA. After adding H_2_SO_4_ into the oil-based solution with 0.1 mmol/L concentration, its peak current of peak I decreased to below the detection limit, whereas its peak II appeared at ~1026 mV and its peak current increased markedly to 53 μA, which can be attributed to a deeper oxidation step in which the phenoxyl radical (PhO•) is further transformed to quinone, i.e., an overall two-electron pathway [31]. Under the acidic conditions, protonation limits O–H dissociation, so the proton-coupled electron transfer (PCET) pathway is suppressed and oxidation shifts to the higher-potential route; as a result, peak II increases while peak I disappears. Under alkaline conditions, the first oxidation peak shifted positively to ~270 mV, with the peak current increasing to 36 μA, whereas the second oxidation peak shifted to lower potential and appeared at ~900 mV, with the peak current decreasing to 9 μA, indicating that the deprotonation is easier, which promotes the PCET step and supports the oxidation reaction in peak I, so peak I becomes dominant [47].

Figure 5b compares the peak current ratio of II to I (Ip2/Ip1) trends of the five phenols such as α-tocopherol, δ-tocopherol, BHT, PG and TBHQ across different ranges of acidic and alkaline addition. Among them, δ-tocopherol appears more stable than α-tocopherol, and Ip2/Ip1 of δ-tocopherol shows a weaker decrease in peak current with increasing solution alkalinity, plausibly because δ-tocopherol with fewer methyl substituents on the chromanol ring than α-tocopherol provides weaker electron donation relatively to α-tocopherol, leading to a smaller pH decrease. Under alkaline conditions, both oxidation peaks of PG disappeared; the second oxidation peak current of BHT decreased and the first peak became broadened; and the two oxidation peaks of TBHQ split into four peaks. Therefore, under alkaline conditions, these three antioxidants are not suitable for comparison based on current ratios. Under acidic conditions, BHT and PG exhibit larger variations in Ip2/Ip1, indicating a stronger preference for the high-potential oxidation pathway and a more pronounced acid–base control. By contrast, Ip2/Ip1 of TBHQ varies least with acid/base addition level because TBHQ bears a strongly electron-donating tert-butyl/hydroquinone relatively to BHT and PG so as to more readily follow PCET, suggesting that both oxidation steps of TBHQ remain electroactive in this acidity/alkalinity conditions and that TBHQ displays the higher PCET competence than BHT and PG.

**Figure 6:**
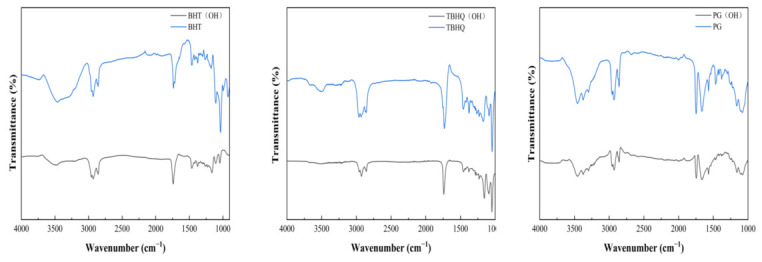
FT-IR spectra of BHT, TBHQ, and PG in the oil-based solution with or without the addition of 10 mmol/L potassium hydroxide.

Notably, the three synthetic phenolic antioxidative substances such as PG, TBHQ and BHT in 0.03–0.18 mmol/L KOH oil-based solution exhibited chromogenic responses. In the MCT-containing supporting electrolyte, 800 mg/kg of PG, TBHQ or BHT exhibited orange, pink, and pale whitish colourations, whereas no colour change was observed for α-tocopherol and δ-tocopherol under the alkaline conditions. These colour changes may be attributed to structural changes in PG, TBHQ, and BHT under alkaline conditions, because phenolic compounds can form phenolate species and absorb visible light at different wavelengths. Similarly, it is reported that gallic acid gradually changes from colourless to brown in alkaline aqueous solution [48]. Correspondingly, the broad absorption bands of TBHQ, PG and BHT under the alkaline conditions in the 3200–3600 cm^−1^ region of FT-IR spectra (Figure 6), assigned to the stretching vibration of phenolic hydroxyl groups, were weakened, indicating that the phenolic hydroxyl groups were deprotonated and converted into phenolate species to some extent. Simultaneously, when assessed by the DPPH radical-scavenging assay, the overall scavenging ratio of PG, TBHQ and BHT with an identical concentration of 150 mg/kg in 10 mL of the oil-based solution after the addition of 0.5 mL of a 10 mmol/L KOH/ethanol solution decreased only slightly by 1–2%. This slight decline can be attributed to the slightly weak antioxidant capacities of the compounds generated from the phenolic antioxidative substances under alkaline conditions, while the majority of the phenolic antioxidative substances remain unoxidized over the timescale of the measurement.

The effects of acid and alkaline content on electrochemical oxidation of β-carotene and lutein were also investigated during the experiments (Appendix A). In the oil-based solution, the voltammetric response of β-carotene and lutein was largely insensitive to acid and alkaline addition. After 60 μL 10 mmol/L H_2_SO_4_ ethanol solution addition, both the peak current and peak potential of β-carotene and lutein in the oil-based solution with 2632 mg/kg of identical concentration were essentially unchanged. After 60 μL 10 mmol/L KOH ethanol solution addition, the overall oxidation peak profile of β-carotene and lutein shifted downward, and the peak potential increased by only approximately 30 mV. This weak acid/base response indicates a non-PCET oxidation pathway for β-carotene and lutein. Because they lack phenolic O–H groups, acid/base addition mainly changes the interfacial environment and solution resistance rather than directly regulating proton transfer, resulting in only minor potential shifts while the peak current remains essentially unchanged [49].

The peak current of 1592 mg/kg ergosterol in 10 mL of the oil-based solution was markedly decreased after the addition of 60 μL 10 mmol/L KOH/ethanol solution or H_2_SO_4_/ethanol solution. This decrease can be attributed to adsorption of oxidation products on the electrode surface, which reduces the effective electroactive area and results in significant current decay [50].

## 4. Conclusions

This study developed an oil-compatible non-aqueous electrochemical platform for evaluating the oxidation behaviour of representative antioxidative substances in an oil-based solution. The mixed C8 MCT oil/organic solvent system improved conductivity and mass transfer while maintaining a low-polarity oil-like environment, enabling the direct characterization of phenolic antioxidants, carotenoids, and ergosterol. The results show that, under the present oil-based conditions, oxidation peak potential served as an empirical indicator of oxidation tendency and was generally associated with radical-scavenging performance, whereas peak current provides information on their electrochemical response intensity and concentration-dependent behaviour. Phenolic antioxidants were mainly governed by proton-coupled electron transfer and secondary quinone/hydroquinone-type oxidation pathways, while carotenoids and ergosterol showed distinct non-phenolic oxidation behaviours. Acid–base regulation further confirmed that proton availability strongly affects the oxidation pathway of phenolic antioxidants. Overall, this work clarifies the relationship among molecular structure, electrochemical oxidation behaviour, and radical-scavenging performance in an oil-based medium, and provides a rapid and practical tool for antioxidant screening, oxidation-property evaluation, oil-quality control, and formulation optimization in edible oil systems.

## Figures and Tables

**Table 1:** Electrochemical parameters for the first oxidation peak of antioxidative substances in the oil-based solution. E_p,1_: potential of the first oxidation peak; I_p,1_: current of the first oxidation peak; all concentrations were 100 mg/kg. The first range in parentheses represents the mass concentration, and the second range represents the corresponding molar-equivalent concentration. Scan rate: 100 mV s^−1^.

Antioxidative Substances	E_p_,_1_ (mV)	I_p_,_1_ (μA)	Concentration–First Oxidation Peak Current Fit (R^2^)
α-Tocopherol	95.00 ± 2.47	3.82 ± 0.24	y = 4.4076 − 0.0013x + 1.7167 × 10^−5^x^2^ − 4.6454 × 10^−9^x^3^
			R^2^ = 0.9974 (100–2600 mg/kg; 0.232–6.032 mmol/kg)
δ-Tocopherol	190.67 ± 2.87	4.33 ± 0.47	y = 0.4552 + 0.0490x − 2.5951 × 10^−5^x^2^ + 4.8290 × 10^−9^x^3^
			R^2^ = 0.9946 (100–2050 mg/kg; 0.248–5.084 mmol/kg)
PG	74.00 ± 2.83	6.00 ± 0	y = 5.40501 − 0.0015x + 8.1218 × 10^−5^x^2^ − 4.7816 × 10^−8^x^3^
			R^2^ = 0.9999 (100–800 mg/kg; 0.471–3.770 mmol/kg)
TBHQ	−247.00 ± 7.87	16.00 ± 0	y = 5.6681 + 0.10671x − 5.8439 × 10^−5^x^2^ − 4.4552 × 10^−10^x^3^
			R^2^ = 0.9995 (80–800 mg/kg; 0.481–4.813 mmol/kg)
BHT	256.33 ± 6.8	6.97 ± 0.19	y = 4.4019 + 0.0267x − 1.2724 × 10^−5^x^2^ + 2.1383 × 10^−9^x^3^
			R^2^ = 0.9992 (100–1800 mg/kg; 0.454–8.169 mmol/kg)
β-Carotene	562.32 ± 1.12	3.42 ± 0.21	y = 2.49ln(x−33.006) − 7.32
			R^2^ = 0.9963 (100–2700 mg/kg; 0.186–5.029 mmol/kg)
Lutein	631.33 ± 13.96	8.41 ± 0.47	y = 0.0284x + 7.3843
			R^2^ = 0.9933 (57–1704 mg/kg; 0.100–2.995 mmol/kg)
ergosterol	1240.42± 4.52	2.32 ± 0.24	y = 0.0425x-2.98
			R^2^ = 0.9893 (100–2000 mg/kg; 0.252–5.042 mmol/kg)

**Table 2:** Electrochemical parameters for the second oxidation peak of antioxidative substances in the oil-based solution. E_p,2_: potential of the second oxidation peak; I_p,2_: current of the second oxidation peak; all concentrations were 1400 mg/kg. The first range in parentheses represents the mass concentration, and the second range represents the corresponding molar-equivalent concentration. Scan rate: 100 mV s^−1^.

Antioxidative Substances	E_p_,_2_ (mV)	I_p_,_2_ (μA)	C–I_p_ Fit (R^2^)
α-Tocopherol	841.33 ± 2.05	5.43 ± 0.05	y = 0.0210x − 23.61
			R^2^ = 0.9961 (1400–5700 mg/kg; 3.250–13.234 mmol/kg)
δ-Tocopherol	990.24 ± 1.41	14.43 ± 0.29	y =0.0279x − 21.4070
			R^2^ = 0.9939 (1150–5800 mg/kg; 2.856–14.405 mmol/kg)
PG	1128.05 ± 19.44	83.33 ± 0.94	y = 0.0701x − 16.31
			R^2^ = 0.9947 (500–2100 mg/kg; 2.356–9.896 mmol/kg)
TBHQ	1062.54 ± 2.83	84.33 ± 0.47	y = 0.09767x − 57.79
			R^2^ = 0.9964 (600–1600 mg/kg; 3.610–9.626 mmol/kg)
BHT	1439.12 ± 3.74	69.87 ± 0.12	y = 0.0520x − 3.63
			R^2^ = 0.9958 (300–1800 mg/kg; 1.361–8.169 mmol/kg)

**Figure A1:**
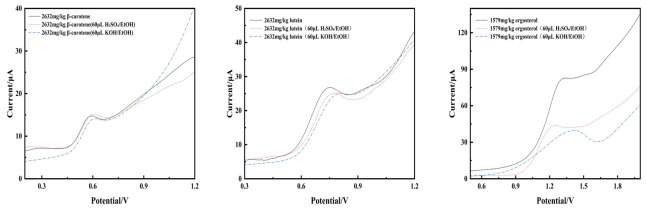
Effects of acidity/alkalinity on the electrochemical properties of β-tocopherol, lutein, and ergosterol.

## Data Availability

The original contributions presented in the study are included in the article. Further inquiries can be directed to the corresponding author.
